# Vandetanib

**Published:** 2012-03-01

**Authors:** J. Michael Vozniak, Joanna M. Jacobs

**Affiliations:** From Hospital of the University of Pennsylvania, Philadelphia, Pennsylvania, and Morristown Medical Center, Morristown, New Jersey

The incidence of thyroid cancer, the most common cancer of the endocrine system, has been steadily increasing since the mid-1990s (American Cancer Society [ACS], 2012). In 2012, it is estimated there will be approximately 56,460 new diagnoses of thyroid cancer (43,210 in women and 13,250 in men) and approximately 1,780 deaths (1,000 women and 780 men) (ACS, 2012). There are three main types of thyroid cancer: differentiated (including papillary, follicular, and Hürthle cell), medullary, and anaplastic.

Medullary thyroid cancer (MTC) accounts for about 4% of all thyroid cancers and is derived from calcitonin-secreting parafollicular C cells (Hundahl et al., 1998). Eighty percent of MTC cases are sporadic and 20% are hereditary (Khan, 2011). The hereditary form of MTC, as well as about 10% of the sporadic form of MTC, results from a mutation in the *RET* (rearranged during transfection) proto-oncogenes (ACS, 2011).

The 10-year overall relative survival rate for MTC is 75% (Hundahl, Fleming, Fremgen, & Menck, 1998). The US Food and Drug Administration (FDA) recently approved vandetanib (Caprelsa) for the treatment of symptomatic or progressive MTC in patients with unresectable locally advanced or metastatic disease (AstraZeneca, 2011).

## Pharmacology

Vandetanib is an oral tyrosine kinase inhibitor shown to have activity against the epidermal growth factor receptor family, vascular endothelial growth factor (VEGF) receptors, RET, protein tyrosine kinase 6 (BRK), tyrosine kinase with immunoglobulin and EGF domains-2 (TIE2), members of the ephrin (EPH) receptor kinase family, and members of the Src family of tyrosine kinases (AstraZeneca, 2011). In vivo vandetanib administration reduced tumor cell–induced angiogenesis, tumor vessel permeability, and inhibited tumor growth and metastasis in mouse models of cancer (AstraZeneca, 2011). Vandetanib has a median plasma half-life of 19 days with steady state achieved at approximately 3 months. Vandetanib has slow oral absorption with peak plasma concentrations achieved at a median of 6 hours. It is 90% bound to alpha-1-acid-glycoprotein and albumin, undergoes hepatic metabolism via cytochrome P450 3A4 (CYP3A4), and is excreted in feces (44%) and urine (25%) (AstraZeneca, 2011).

Vandetanib is available in 300- and 100-mg tablets. The recommended daily dose of oral vandetanib is 300 mg once daily. Vandetanib may be taken with or without food. If a patient misses a dose, the missed dose should not be taken if it is less than 12 hours before the next dose. For patients who have difficulty swallowing the vandetanib capsules whole, the capsules should not be crushed. The tablets can be dispersed in a glass containing 2 ounces of noncarbonated water and stirred for approximately 10 minutes until the tablet is dissolved; the mixture should be swallowed immediately. See the package insert for more information (AstraZeneca, 2011). Table 1 provides dosage recommendations for patients with renal or hepatic impairment and those in whom QTcF (QT interval with Fridericia correction) prolongation or other toxicities occur. Due to metabolism via CYP3A4, use of concomitant strong inducers and inhibitors of CYP3A4 should be avoided. However, no interaction was found between itraconazole, a strong CYP3A4 inhibitor, and vandetanib in healthy subjects. The risk of drug interactions with other CYP3A4 inhibitors is unknown, so precautions should be taken. In addition, drugs known to prolong the QT interval should be avoided while taking vandetanib (Table 2).

**Table 1:**
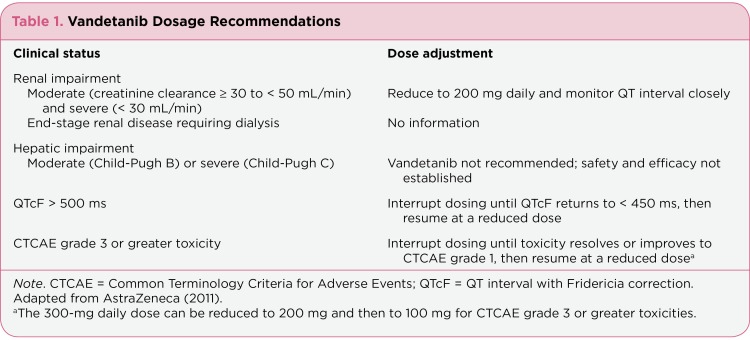
Table 1. Vandetanib Dosage Recommendations

**Table 2:**
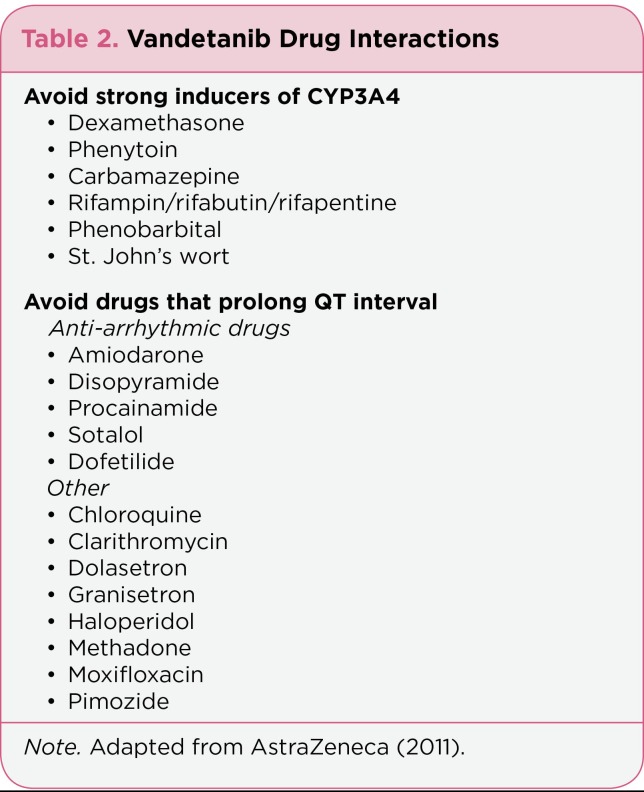
Table 2. Vandetanib Drug Interactions

## Key Clinical Studies

Two open-label, single-arm, phase II studies were conducted to assess the efficacy of vandetanib in adult patients with unresectable locally advanced or metastatic MTC. In both studies, patients with brain metastases were included if they had been treated with radiation therapy or were clinically stable without corticosteroid treatment for 1 week (Wells et al., 2010; Robinson, Paz-Ares, Krebs, Vasselli, & Haddad, 2010).

In the study conducted by Wells et al. (2010), 30 patients received oral vandetanib 300 mg daily. Of those patients, 29 (97%) had distant metastases. The objective response rate (ORR) was 20% (95% confidence interval [CI] = 8%–39%); all responses were partial (PR). Stable disease (SD) = 24 weeks was achieved in 16 (53%) patients with an observed disease control rate (DCR), defined as PR + SD = 24 weeks, of 73% (95% CI = 54%–88%). The median duration of response was 10.2 months (range, 1.9 to 16.9 months; 95% CI = 8.0–13.2 months), and progression-free survival (PFS) at the time of data cutoff was 27.9 months (95% CI not estimable). Reductions in calcitonin and carcinoembryonic antigen (CEA) levels ranged from 73% to 99% and 82% to 91%, respectively. The most common adverse events observed were diarrhea (70%), rash (67%), fatigue (63%), and nausea (63%). Dosage reductions or interruptions occurred in 24 patients, most commonly due to diarrhea (Wells et al., 2010).

In the study conducted by Robinson et al. (2010), 19 patients received oral vandetanib 100 mg daily; 18 (95%) of these patients had metastatic disease. Four patients increased their dose to 300 mg daily after disease progression. The ORR (all partial responses) was 16% (95% CI = 3.4%–39.6%). Stable disease for 24 weeks or longer was observed in 10 (53%) patients, yielding a DCR of 68% (95% CI = 43.4%–87.4%). Reductions in calcitonin levels ranged from 1.4% to 89.4% and met a biochemical PR in 3 (16%) patients. Reductions in CEA levels ranged from 6.9% to 87.9%. Diarrhea (47%), fatigue (42%), rash (26%), and constipation (26%) were the most commonly reported adverse events (Robinson et al., 2010).

In a phase III, randomized, double blind, placebo-controlled trial, 331 patients with unresectable, measurable, locally advanced or metastatic, hereditary or sporadic MTC were treated with either vandetanib 300 mg (n = 231) or placebo (n = 100) daily. Most of the patients in both the vandetanib (94%) and placebo (97%) arms had metastatic disease, of which 87% and 92% involved two or more organs, respectively. The majority of patients (61% and 58%, respectively) had never received systemic therapy for the treatment of their MTC. A statistically significant difference in PFS was observed favoring vandetanib (hazard ratio 0.46, 95% CI = 0.31–0.69; *p* < .001). The median PFS in the vandetanib group had not been reached at the time of analysis, but was estimated to be 30.5 months compared with 19.3 months in the placebo group. In addition, ORR (vandetanib 45%; placebo 13%; odds ratio 5.48, 95% CI = 2.99–10.79; *p* < .001) and DCR (vandetanib 87%; placebo 71%; odds ratio 2.64, 95% CI = 1.48–4.69; *p* = .001) were both favored in the vandetanib group.

The median duration of treatment was 90.1 weeks with vandetanib and 39.9 weeks with placebo. The most common adverse events occurring in the vandetanib and placebo arms were diarrhea (56% vs. 26%), rash (45% vs. 11%), nausea (33% vs. 16%), and hypertension (32% vs. 5%), with 12% and 3% of patients, respectively, discontinuing therapy due to intolerable toxicity (Wells et al., 2012).

Vandetanib is one of many tyrosine kinase inhibitors being studied in MTC. Sorafenib (Nexavar) has been shown to reduce symptoms due to metastases and elevated calcitonin levels (Kober, Hermann, Handler, & Krotla, 2010). In addition, motesanib (AMG-706)—an investigational agent that is an antagonist of VEGF receptors 1, 2, and 3; platelet-derived growth factor receptor; and c-kit (stem cell factor receptor)—has proven to be effective with a stable disease rate of 82% and a median PFS of 48 weeks (Schlumberger et al., 2009). A number of phase II and III trials studying sorafenib, sunitinib (Sutent), cabozantinib (XL184), and pazopanib (Votrient) in advanced medullary carcinoma (National Institutes of Health [NIH], 2012) are under way. Vandetanib is being studied as combination therapy with the proteasome inhibitor bortezomib (Velcade) in patients with locally advanced or metastatic MTC (NIH, 2012).

## Adverse Events

The most commonly reported (> 20%) adverse events that have occurred in patients taking vandetanib include diarrhea, rash, acne, nausea, hypertension, fatigue, decreased appetite, and abdominal pain (Table 3). Laboratory abnormalities such as hypocalcemia, hypoglycemia, and elevated alanine aminotransferase can also occur. Practitioners should consider monitoring these parameters more closely if they develop or if there is a concern regarding potential development (AstraZeneca, 2011).

**Table 3:**
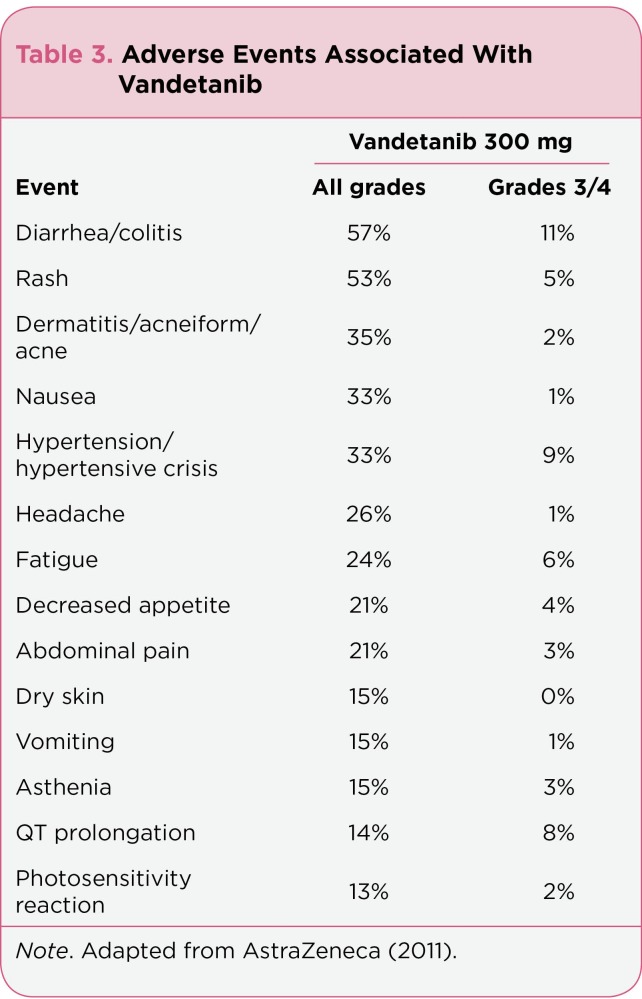
Table 3. Adverse Events Associated With Vandetanib

In clinical trials, vandetanib caused QT prolongation in 14% of patients. The drug should not be initiated in patients with a QTc interval greater than 450 ms or given to patients with a history of torsades de pointes, congenital long-QT syndrome, bradyarrhythmias, or uncompensated heart failure. Vandetanib has also been associated with photosensitivity and serious skin reactions, such as Stevens-Johnson syndrome. Patients should be advised to wear sunscreen during vandetanib therapy and for up to 4 months after completion of treatment. Mild to moderate skin reactions can be managed with topical or systemic corticosteroids, oral antihistamines, or topical or systemic antibiotics (AstraZeneca, 2011).

Other rare serious adverse reactions reported with vandetanib include interstitial lung disease, ischemic cerebrovascular events, serious hemorrhagic events, heart failure, hypothyroidism, hypertension (including hypertensive crisis), and reversible posterior leukoencephalopathy syndrome. Adverse reactions to vandetanib may not resolve quickly due to its long half-life of 19 days (AstraZeneca, 2011).

## Role in Therapy

Surgery is the current standard treatment for patients with newly diagnosed, localized MTC. Radioactive iodine therapy is not recommended for the treatment of MTC because MTC cells do not accumulate radioiodine (National Comprehensive Cancer Network [NCCN], 2011; Kloos et al., 2009).

Very few treatment options are available for patients with locally advanced, metastatic, or recurrent MTC. Prior to the FDA approval of vandetanib, treatment options included surgical resection, external beam radiation therapy, dacarbazine-based chemotherapy, regional ablation via radiofrequency or embolization, or enrollment in a clinical trial (NCCN, 2011; Kloos et al., 2009). No treatment strategy was clearly favored due to the minimal benefit gained; enrollment in a clinical trial was preferred. Vandetanib is now a viable treatment option for patients with locally advanced, metastatic, or recurrent MTC. It is the first tyrosine kinase inhibitor to be approved for this indication after showing a beneficial response rate and improved PFS over placebo in a phase III randomized clinical trial (Wells et al., 2012).

Given its manageable adverse event profile, vandetanib is a reasonable treatment option in patients with progressed MTC. It is recommended that vandetanib be utilized in patients with metastatic, recurrent, or persistent disease that has become symptomatic or is growing (NCCN, 2011). Therapy should be continued until disease progression or until patients have intolerable toxicity from treatment (AstraZeneca, 2011). Vandetanib should not be started in asymptomatic patients with detectable elevations of their laboratory markers, such as calcitonin or CEA (NCCN, 2011).

## Implications for the Advanced Practitioner

Patients being considered for treatment with vandetanib should have the following baseline parameters checked: electrocardiogram (ECG), serum potassium, calcium, magnesium, and thyroid-stimulating hormone. These parameters should be checked again at 2 to 4 weeks, at 8 to 12 weeks, and every 3 months routinely after starting treatment with vandetanib. Serum potassium levels should be maintained at 4 mEq/L or higher, and serum magnesium and serum calcium should be kept within the normal range to reduce the risk of QT prolongation.

Diarrhea is one of the main adverse reactions associated with vandetanib. Due to the risk of diarrhea causing electrolyte imbalances and vandetanib’s risk of QT prolongation, patients should be counseled on the appropriate use of antidiarrheal agents to prevent more serious complications. Serum electrolytes and ECGs should be monitored in patients who experience diarrhea. If severe diarrhea develops, vandetanib should be discontinued until diarrhea improves.

Approximately one-third of patients taking vandetanib develop hypertension or hypertensive crisis. Patients should be routinely checked for the development of hypertension and encouraged to monitor their own blood pressure. Hypertension should be controlled as appropriate; dose reductions or interruptions may be necessary. In patients who develop high blood pressure that cannot be controlled, vandetanib should be discontinued.

Although the incidence of pulmonary toxicities (interstitial lung disease and pneumonitis) and heart failure are low, deaths have been reported in patients taking vandetanib. Patients should be monitored for signs and symptoms of heart failure and pulmonary toxicities.

Due to the risk of QT prolongation, torsades de pointes, and sudden death, the FDA has required the implementation of a Risk Evaluation and Mitigation Strategy (REMS) program. Under the Caprelsa REMS program, only prescribers and pharmacies enrolled in the program can prescribe and dispense vandetanib (AstraZeneca, 2011). In order to prescribe vandetanib, prescribers must read a Healthcare Provider (HCP) letter; review a HCP Education Pamphlet or HCP REMS Education Slide Set and the vandetanib full prescribing information; complete the Prescriber Training Program; and complete the Prescriber Enrollment form. Vandetanib is not available at retail pharmacies and can only be dispensed through a restricted distribution program managed by Biologics, Inc. Individual patient prescription forms must be completed and submitted to Biologics, Inc., which then reviews insurance coverage and arranges drug delivery to the patient. Enrollment information is available at www.caprelsarems.com

## Summary

Vandetanib is a well-tolerated oral agent for the treatment of symptomatic or progressive MTC in patients with unresectable locally advanced or metastatic disease that has demonstrated improved PFS compared to placebo. Due to the risk of QT prolongation, torsades de pointes, and sudden death, prescribers must enroll in the Caprelsa REMS program in order to prescribe vandetanib. Vandetanib requires routine monitoring of ECGs and electrolytes and careful observation for the development of other toxicities.
